# Anterior retropharyngeal cage distraction with atlantoaxial plate-screw fixation for basilar invagination with atlantoaxial dislocation

**DOI:** 10.3171/2020.4.FocusVid.20157

**Published:** 2020-07-01

**Authors:** Sushil Patkar

**Affiliations:** Department of Neurosurgery, Poona Hospital and Research Center, Pune, Maharashtra, India

**Keywords:** basilar invagination, atlantoaxial dislocation, atlantoaxial fixation

## Abstract

The unilateral submandibular anterior retropharyngeal approach in properly selected patients offers the possibility to expose both atlantoaxial joints adequately, abrade the endplates, and graft the joint spaces. The supine position in extension permits the use of wedge-shaped cages, which reduce the invagination and correct the dislocation. Adequate bone stock is available to rigidly fix the joints using an anterior plate-screw construct without any risk to the vertebral arteries. The approach preserves the posterior tension band and the C2 root. The technique is quick, simple, and safe, and results in solid fusion of the joints over time.

The video can be found here: https://youtu.be/tT6j3Czy6tc

## Transcript

**0:20** Introduction and evolution of the concept. Anterior cage distraction for basilar invagination with atlantoaxial dislocation. Posterior distraction and atlantoaxial fixation has become a standard for treatment of basilar invagination with atlantoaxial dislocation. Posterior surgery has many problems. Vertebral artery injury always remains a potential problem. Anterior surgery eliminates almost all the risk of posterior surgery. The wedge cage can reposition the dislocated atlas upward and backward. The supine position with dissection between the muscle planes and only a small amount of disruption of the longus colli helps in preservation of the posterior tension band. Correction of the cervicomedullary strain is the aim of this new operation (Patkar, 2013).

**1:27** Concept, animation, and cadaveric dissection. The wedge-shaped cage pushes the atlas upward and backward. The large lateral mass and the body of C2 offer enough bone stock for rigid fixation in the reduced position. The supine position with extension helps in reducing the atlantoaxial dislocation. The incision, one finger breadth the angle of the mandible, avoids injury to the marginal mandibular nerve. The hypoglossal nerve is the only important structure which needs to be protected and acts as a landmark to reach the prevertebral space. In this cadaveric dissection, after dividing the platysma, digastric muscle is easily visible. The digastric muscle is exposed all throughout its length. The submandibular gland lies superiorly above the digastric tendon. This is the stylohyoid muscle below which is the hypoglossal nerve. Gently dissecting with your finger below the hypoglossal nerve and medial to the carotid artery gives easy access to the prevertebral space onto the anterior surface of the axis. Thin, long Langenbeck retractors are very handy in working at depth. Both the atlantoaxial joints, on either side of the midline, are visible. Opening the joints, gardening the surfaces, and putting bone grafts is an integral part of this operation (Patkar, 2015, 2016).

**4:10** Live surgical demonstration. These are the two longus colli muscles, this is the midline, and the atlantoaxial joints are just under the longus colli. Using a sheathed cautery to avoid injury to surrounding structures, the medial portion of the longus colli is divided to expose the atlantoaxial joints on either side of midline. The anterior permits a wide exposure of the atlantoaxial joint, the lateral mass of the atlas from side to side, and the anterior surface of the axis without and bleeding from the paravertebral venous plexus or the vertebral artery. The joints are prepared by passing the microdrill up to a depth of 1 cm, and the endplates are abraded, curetted till there is bleeding bone, which is so essential for getting good fusion. Time spent over here helps in the final result, that is, fusion. Corticocancellous bone harvested from the anterior iliac crest is packed into the joint to provide for osteogenesis and osteoinduction. The cage packed with bone graft is impacted gradually into the joint space, and the plate is fixed across the joint by passing one screw in the lateral mass of C1, upward and outward, and another screw into the body of axis, downward and medially, without any risk of injury to the vertebral artery (Menon and Raniga, 2018).

**6:55** Long-term results and references. This is an example of anterior surgery with long-term result showing rigid fusion. Cages up to 9 mm can be used to achieve distraction and realignment (Patkar, 2019; Menon 2019).

Thank you.
